# Ex vivo cortical porosity and thickness predictions at the tibia using full-spectrum ultrasonic guided-wave analysis

**DOI:** 10.1007/s11657-019-0578-1

**Published:** 2019-02-20

**Authors:** Johannes Schneider, Gianluca Iori, Donatien Ramiandrisoa, Maroua Hammami, Melanie Gräsel, Christine Chappard, Reinhard Barkmann, Pascal Laugier, Quentin Grimal, Jean-Gabriel Minonzio, Kay Raum

**Affiliations:** 1 Berlin-Brandenburg School for Regenerative Therapies (BSRT), Charité - Universitätsmedizin Berlin, Augustenburger Platz 1, 13353 Berlin, Germany; 2 Laboratoire d’Imagerie Biomédicale (LIB), CNRS, INSERM, Sorbonne University, 15, rue de l’école de médecine, 75006 Paris, France; 3 BleuSolid, 29, rue Beauséjour, 77400 Pomponne, France; 4 Molecular Imaging North Competence Center (MOIN), Universitätsklinikum Schleswig-Holstein, Am Botanischen Garten 14, 24118 Kiel, Germany; 5 Osteo-Articular Bioengineering and Bioimaging (B2OA), CNRS, INSERM, University Denis Diderot, 10, Avenue de Verdun, 75010 Paris, France; 6 Escuela de Ingeniería Civil en Informática, Universidad de Valparaíso, General Cruz 222, Valparaíso, Chile

**Keywords:** Acoustic microscopy, Axial transmission ultrasound, Cortical bone porosity, Guided waves, Micro-computed tomography

## Abstract

**Summary:**

The estimation of cortical thickness (Ct.Th) and porosity (Ct.Po) at the tibia using axial transmission ultrasound was successfully validated ex vivo against site-matched micro-computed tomography. The assessment of cortical parameters based on full-spectrum guided-wave analysis might improve the prediction of bone fractures in a cost-effective and radiation-free manner.

**Purpose:**

Cortical thickness (Ct.Th) and porosity (Ct.Po) are key parameters for the identification of patients with fragile bones. The main objective of this ex vivo study was to validate the measurement of Ct.Po and Ct.Th at the tibia using a non-ionizing, low-cost, and portable 500-kHz ultrasound axial transmission system. Additional ultrasonic velocities and site-matched reference parameters were included in the study to broaden the analysis.

**Methods:**

Guided waves were successfully measured ex vivo in 17 human tibiae using a novel 500-kHz bi-directional axial transmission probe. Theoretical dispersion curves of a transverse isotropic free plate model with invariant matrix stiffness were fitted to the experimental dispersion curves in order to estimate Ct.Th and Ct.Po. In addition, the velocities of the first arriving signal (*υ*_*FAS*_) and A_0_ mode (*υ*_*A0*_) were measured. Reference Ct.Po, Ct.Th, and *vBMD* were obtained from site-matched micro-computed tomography. Scanning acoustic microscopy (SAM) provided the acoustic impedance of the axial cortical bone matrix.

**Results:**

The best predictions of Ct.Po (R^2^ = 0.83, RMSE = 2.2%) and Ct.Th (R^2^ = 0.92, RMSE = 0.2 mm, one outlier excluded) were obtained from the plate model. The second best predictors of Ct.Po and Ct.Th were *vBMD* (R^2^ = 0.77, RMSE = 2.6%) and *υ*_*A0*_ (R^2^ = 0.28, RMSE = 0.67 mm), respectively.

**Conclusions:**

Ct.Th and Ct.Po were accurately predicted at the human tibia ex vivo using a transverse isotropic free plate model with invariant matrix stiffness. The model-based predictions were not further enhanced when we accounted for variations in axial tissue stiffness as reflected by the acoustic impedance from SAM.

**Electronic supplementary material:**

The online version of this article (10.1007/s11657-019-0578-1) contains supplementary material, which is available to authorized users.

## Introduction

In postmenopausal women, the majority of bone is lost from the cortical bone compartment as a result of both reduced cortical thickness (Ct.Th) and increased cortical porosity (Ct.Po) [1]. Both parameters can be measured in vivo with high-resolution peripheral quantitative computed tomography (HR-pQCT) and were recently shown to be associated with a higher prevalence of hip fractures [2]. However, this novel imaging technology is still rarely available and based on ionizing radiation. Alternatively, quantitative ultrasound (QUS) techniques are being developed, which are non-ionizing, low cost, and portable. For example, a simple ultrasonic pulse-echo measurement was proposed to predict Ct.Th, but the ultrasonic wave-speed in the cortical bone layer was assumed to be known [3].

Modern ultrasound axial transmission (AT) measures the dispersion curves of guided waves, which propagate in the cortical shell of long bones [4]. In early AT applications, isotropic tube models were fitted to the dispersion curves, providing Ct.Th ex vivo at the human radius [5] and bovine tibia [6]. Subsequently, a transverse isotropic free plate model was proposed, the use of which allowed estimating Ct.Th and four bone elastic coefficients at the same time [7]. The unknown coefficients of this plate model were then reduced to Ct.Th and Ct.Po [8]. To build such a model, asymptotic homogenization [9] has been applied to estimate the effective stiffness tensor as a function of porosity, assuming an invariable stiffness of the tissue matrix. However, the mineralization of the bone tissue matrix in humans, intimately related to the stiffness, is not constant, but affected by age [10], gender [11], treatment, and disease [12].

In the beginning of its development, AT has extensively been used to measure the first arriving signal velocity (*υ*_*FAS*_) in the cortex of the radius and tibia. The first arriving signal measured at low frequencies has a larger penetration depth than at high frequencies. Thus, it can capture features of deeper cortical bone layers in which disease-associated changes usually start to occur [13]. Accordingly, low-frequency *υ*_*FAS*_ (200 kHz) measured at the tibia was significantly correlated with Ct.Th (R = 0.24, *p* < 0.001), whereas high-frequency *υ*_*FAS*_ (1.25 MHz) was not [14]. The ability of *υ*_*FAS*_ to discriminate subjects with osteoporotic fractures from non-fractured controls was shown to be similar [15] or inferior [16, 17] when compared to areal bone mineral density (*aBMD*) measured by dual-energy X-ray absorptiometry (DXA) at the hip or spine. DXA is considered the current standard method for osteoporosis diagnosis and fracture risk prediction.

In an attempt to provide complementary parameters to *υ*_*FAS*_ with improved fracture discrimination ability, researchers also considered the phase velocity of the A_0_ mode (*υ*_*A0*_) [18, 19]. A_0_ is a fundamental flexural guided wave, which propagates within the cortical bounds and is particularly sensitive to both Ct.Th and to pathological changes in the endosteal region depending on the frequency-thickness ratio regime [20]. Following these findings, an ex vivo study at the radius showed significant correlations of *υ*_*A0*_ with Ct.Th (R^2^ = 0.52, *p* < 0.001) and with volumetric bone mineral density (*vBMD*) (R^2^ = 0.45, *p* < 0.001) [21]. However, when investigated in vivo at the tibia, the correlations between both *υ*_*A0*_ and *vBMD* and *υ*_*A0*_ and Ct.Th were less strong [14]. According to the authors, the correlations decreased due to interferences with the soft tissue, in which ultrasound propagates at similar velocities (~ 1500 m^.^s^−1^) as the A_0_ mode in cortical bone [22].

In this ex vivo study, we predicted Ct.Th and Ct.Po at the human tibia using a model-based inversion method which was previously proposed by our group for a similar 1-MHz radius probe [23]. To account for the difference in Ct.Th between the tibia and radius, a novel probe was designed to optimize the excitation of guided waves in the Ct.Th range usually found in humans at the diaphysis of the tibia. Compared to the former radius probe, the central frequency is reduced from 1.0 to 0.5 MHz, whereas the probe dimensions are slightly increased. Cortical bone samples were extracted from the region below the receiver array for *Ct.Po*_μCT_ reference measurements using high-resolution micro-computed tomography (μCT, 7.4 μm isotropic voxel size). Site-matched reference *Ct.Th*_*μCT*_ and *vBMD* were obtained from a larger μCT scan at lower resolution (39 μm isotropic voxel size). In addition, we assessed the acoustic impedance (a surrogate for matrix stiffness) using scanning acoustic microscopy (SAM) to evaluate the assumption of a waveguide model with invariant matrix stiffness. The ultrasonic velocities *υ*_*FAS*_ and *υ*_*A0*_ were measured and compared to site-matched cortical bone properties.

## Methods

### Bone samples

Nineteen left tibia specimens without soft tissue from human cadavers (6 male, 13 female, age 69–94 years, mean 83.7 ± 8.4 years) were provided by the Institute of Anatomy, University of Lübeck. The sample collection was obtained in accordance with the German law “*Gesetz über das Leichen-, Bestattungs- und Friedhofswesen des Landes Schleswig-Holstein, Abschnitt II, § 9 Leichenöffnung, anatomisch*” from 04.02.2005. All specimens were received without distal ends (cut off at approximately 50%) and stored at − 20 °C.

### Axial transmission ultrasound

#### Experimental system

The axial transmission (AT) system (Azalée, Paris, France) included a custom-made probe (Vermon, Tours, France), driving electronics (Althais, Tours, France), and a human-machine interface (BleuSolid, Paris, France). The multi-channel probe consisted of a central 24-receiver array (pitch = 1.2 mm) and two lateral 5-emitter arrays (pitch = 1.5 mm). The dimensions of each rectangular receiver and emitter element were 1.2 × 13 mm^2^ and 1.5 × 13 mm^2^, respectively. A distance of 8 mm separated the receiver from each emitter array. This configuration enabled the propagation of ultrasound in two opposite directions, a technique used to correct errors induced by small inclination angles between the probe and the bone surface [24]. The excitation signal consisted of a wideband pulse with a center frequency of 500 kHz (− 6 dB frequency bandwidth from 0.3 to 0.7 MHz). The five multi-element transmitters were used successively with time delays ranging from 0 to 0.8 μs. After 16 averages performed by the hardware, a set of 120 radio-frequency (RF) signals corresponding to all possible transmission-receiver pairs were digitized (12 bits, 20 MHz, 1024 samples) for each propagation direction.

#### Measurement protocol

**Fig. 1:**
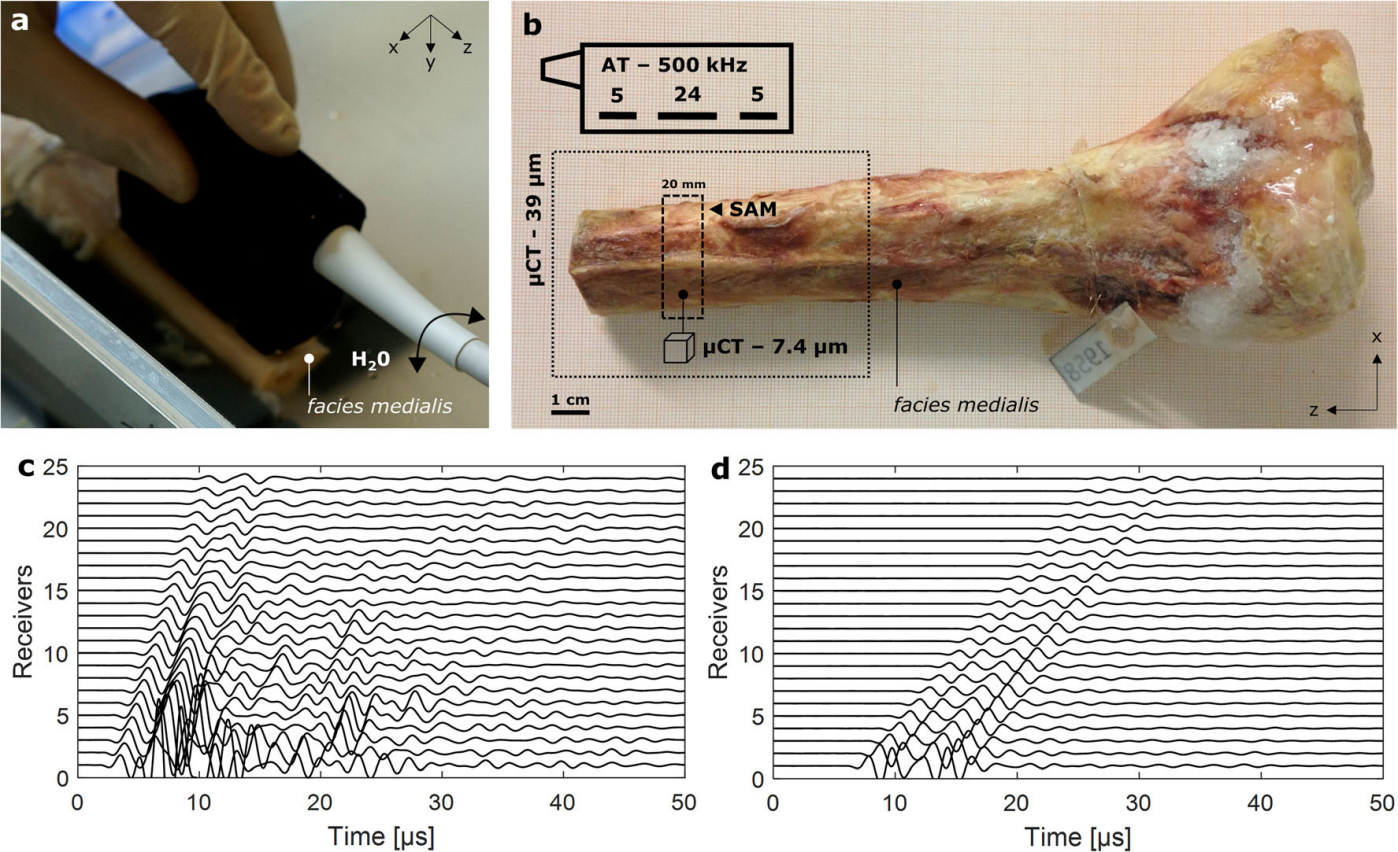
**a** 500-kHz axial transmission (AT) multi-channel probe positioned on the *facies medialis* and aligned with the z-axis of a tibia specimen. The arrow indicates the movement of the probe during the acquisition of 400 individual measurements. **b** *Top left* sketch of probe showing the number and positions of central receivers and adjacent lateral emitters. The distal ends of each tibia (pointed line box) were imaged using micro-computed tomography (μCT, 39 μm isotropic voxel size). A cross-section (dashed line box) was extracted from the AT measurement region. The proximal surface of the cross-section was scanned with 100-MHz scanning acoustic microscopy (SAM). A parallelepiped sample of around 2 × 3 × 4 mm^3^ was obtained from the *facies medialis* of this cross-section and imaged with μCT (7.4 μm isotropic voxel size). Typical waveforms acquired after one ultrasound transmission at the tibia ex vivo (**c**) and in a water tank of 65 mm depth (**d**)

Measurements were performed in water at room temperature (21 °C) (Fig. 1a). The probe was placed in contact with the specimens at the medial surface of the tibia (*facies medialis*) above the medullary cavity. The edge of the probe was aligned with (i) the distal cut plane and (ii) the long main axis of the bone. The protocol required the acquisition of three cycles of 400 successive measurements. During each cycle, the probe was slowly tilted in both circumferential directions (arrow in Fig. 1a) to scan a wide region above the medullary cavity. Between the cycles, the probe was removed from the water bath and repositioned. At each measurement, 120 RF signals (5 × 24) were acquired from each propagation direction. The scan time per cycle was about 3 min.

#### Cortical porosity and thickness estimation

**Fig. 2:**
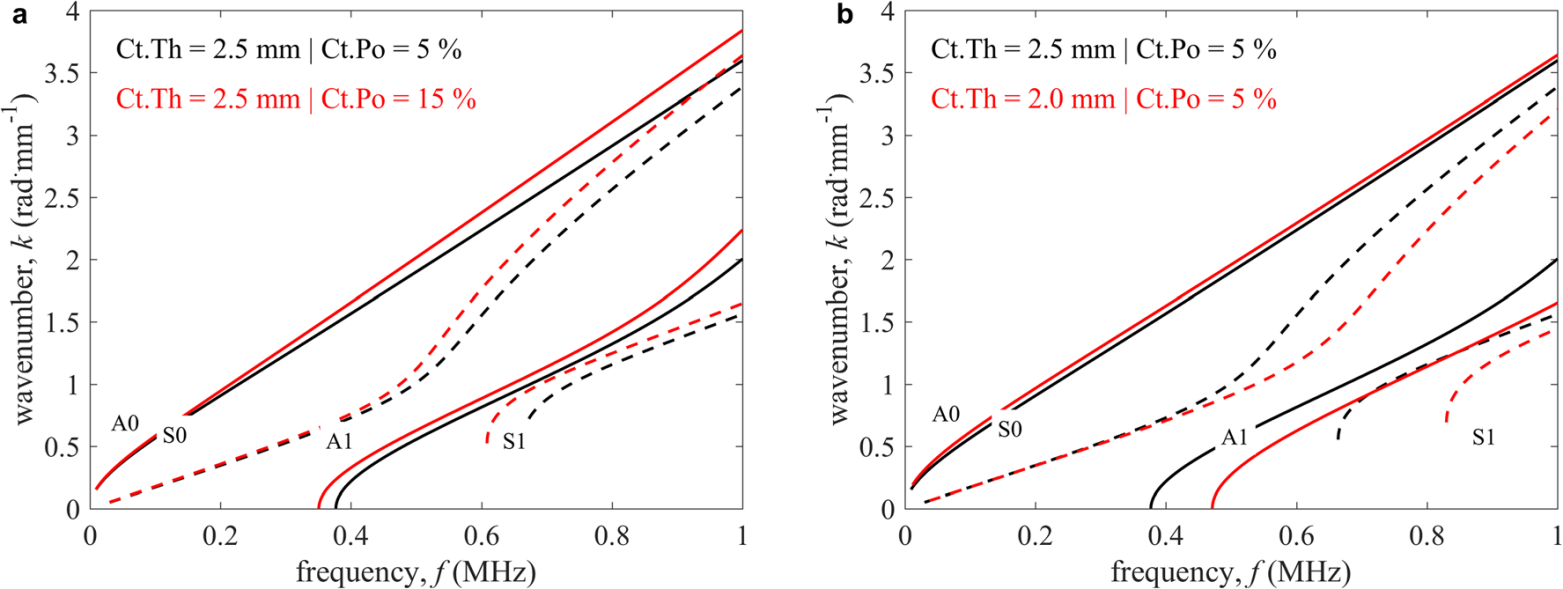
Dispersion curves of the transverse isotropic free plate model with homogenized elastic properties in the frequency-wavenumber (*f*-*k*) space. **a** Constant cortical thickness (Ct.Th) (2.5 mm) with varying cortical porosity (Ct.Po) (5 and 15%). **b** Constant Ct.Po (5%) with varying Ct.Th (2.5 and 2.0 mm). Antisymmetric (A) and symmetric (S) modes are represented as continuous and dashed lines, respectively

**Fig. 3:**
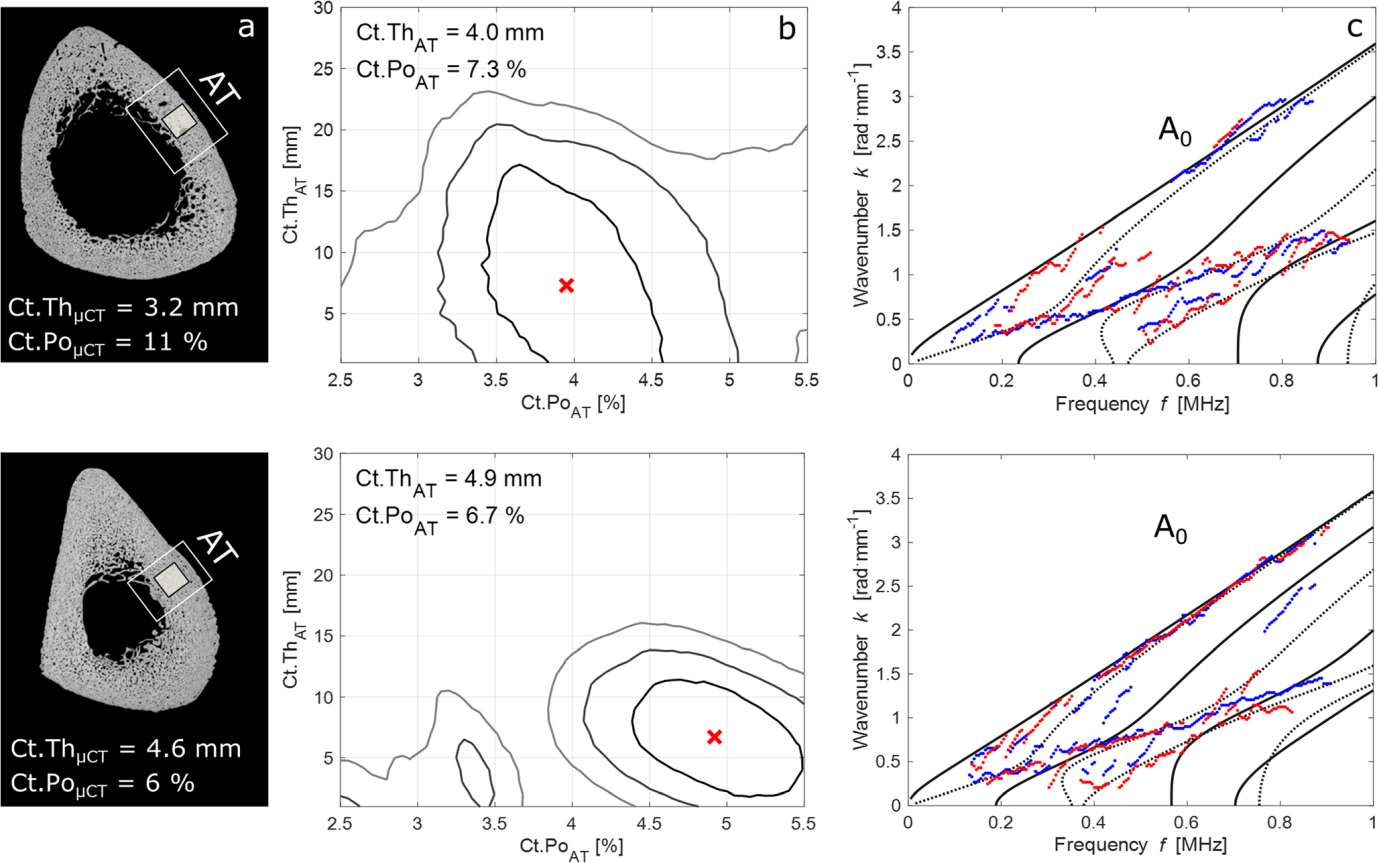
**a** 100-MHz scanning acoustic microscopy (SAM) images with 12 μm pixel size, showing the axial transmission (AT) measurement region. The cortical thickness (Ct.Th) below the probe was measured using μCT with 39 μm voxel size. The black square indicates the region from which a parallelepiped sample was extracted for cortical porosity (Ct.Po) measurements using μCT with 7.4 μm voxel size. **b** Contour plot representations of the objective functions with global maxima (crosses) corresponding to the best fit between the waveguide model and experimental dispersion curves (**c**). Continuous and dashed lines represent antisymmetric and symmetric modes, respectively. Red and blue dotted lines correspond to the experimental dispersion curves obtained from bi-directional guided wave measurements

Cortical thickness (Ct.Th) and porosity (Ct.Po) were estimated by fitting a transverse isotropic free plate model (Fig. 2) to the measured guided wave dispersion curves in line with Minonzio et al. [23]. Briefly, the recorded time signals were transformed to the frequency-wavenumber (*f*-*k*) space using a singular value decomposition (SVD) enhanced two-dimensional spatiotemporal Fourier transform. This signal processing step provided the so-called *Norm function* of which each pixel (*f*, *k*) reflects the presence rate of a guided wave mode in a 0 to 1 scale [4]. Subsequently, a transverse isotropic free plate model was fitted to the maxima of the *Norm function* (Fig. 3c). A plate model was used since effects from the bone’s curvature can be neglected [25]. The model required four elastic coefficients, the mass density, and the thickness of the waveguide. Granke et al. suggested that in aged women, changes in Ct.Po account for most of the bone’s mesoscopic elasticity variations [26]. We used thus a micro-mechanical model [9] to calculate a set of effective mesoscale stiffness tensors for a set of porosity values assuming that transverse isotropic elastic coefficients (*c*_*11*_ = 26.8 GPa, *c*_*13*_ = 15.3 GPa, *c*_*33*_ = 35.1 GPa, and *c*_*55*_ = 7. 3 GPa) and mass density (*ρ* = 1.91 g^.^cm^−3^) for the tissue matrix are invariant [26]. Then, the predicted mesoscale stiffness tensors were used to create a database of dispersion curves for a combination of porosity and thickness values. The thickness ranged from 2.5 to 5.5 mm with intervals of 0.1 mm and the porosity from 1 to 25% with intervals of 1%. Figure 2 shows the effect of changes in Ct.Th and Ct.Po on the modeled dispersion curves. Ct.Po mainly modifies the slope of the curves in the *f*-*k* space (a), whereas the curves shift towards lower frequencies with increasing Ct.Th (b).

To find the best fit between the plate model and the experimental dispersion curves (Fig. 3c), the model database was projected onto the singular vector basis *U(f)* of the *Norm function*. Accordingly, the objective function is denoted as

1 $$ Proj\left( Ct. Th, Ct. Po\right)=\frac{1}{fmax- fmin}{\int}_{fmin}^{fmax}{\sum}_{m=1}^M{\left\Vert {e}^{test}\left( km\left(f, Ct. Th, Ct. Po\right)\right)\right\Vert}_{U(f)}^2 df, $$

where *f*_*min*_ and *f*_*max*_ correspond to the frequency bandwidth limits, *M* denotes the number of theoretical guided modes, and *e*^*test*^ the testing vector being a normalized attenuated plane wave. Figure 3b shows contour plot representations of the objective function with the global maxima corresponding to the fitted models in Fig. 3c. Due to ill conditioning of the objective function, i.e., incomplete experimental dispersion curves, often more than one local maxima was obtained. To remove this model ambiguity, we compared the two highest local maxima for each of the 400 successive measurements: when the highest maximum (global) exceeded the second highest of at least 3%, this was considered to be a valid solution to the problem. The threshold was empirically chosen based on a tradeoff between the standard deviation of the Ct.Th/Ct.Po estimates and the total number of valid measurements per measurement cycle. If at least 10 of the 400 successive measurements produced a valid parameter pair, the medians of the Ct.Th/Ct.Po estimates were calculated. Otherwise, the entire measurement cycle would have been rejected. Finally, if at least two out of three cycles were valid, the medians of these valid cycles were averaged for every specimen. Otherwise, the entire measurement series for that sample would have been rejected.

#### Estimation of the first arriving signal velocity

The velocity of the first arriving signal (*υ*_*FAS*_) was calculated based on a bi-directional measurement [27]. Briefly, the time of flight was determined for each emitter-receiver distance using the first extremum of the signal in the time domain. The sound velocity was derived from the inverse slope of a linear fit through these time points plotted against the known emitter-receiver distances. This procedure was performed for each of the five transmissions and from both directions to account for small inclination angles between the probe and the bone surface. It was previously shown that larger probe inclination angles increase the relative measurement error of *υ*_*FAS*_ [28]. Accordingly, bi-directional measurements for which the absolute difference between the two opposite velocities exceeded 50 m s^−1^ were eliminated [29]. Ideally, a measurement provided five corrected velocities, corresponding to the five bi-directional ultrasound transmissions per measurement, which were then averaged. Histograms of the corrected velocities were obtained for each measurement cycle. For each specimen, the *υ*_*FAS*_ was calculated as the average of the three histogram peaks.

#### Estimation of the A_0_ mode velocity

The A_0_ mode phase velocity (*υ*_A0_) was calculated in the frequency-domain based on SVD-enhanced 2D Fourier transforms of the acquired multi-dimensional radiofrequency signals. The principal signal processing steps are illustrated in Online Resource 1. First, the *Norm function* was converted from the frequency-wavenumber (*f*-*k*) into the frequency-phase velocity (*f*-*cp*) domain (*cp* = 2π *f*/*k*) [30]. Afterwards, the A_0_ mode was extracted using a fixed frequency (0.5 to 0.8 MHz) and *c*_*p*_ range (1400 to 1900 m s^−1^). Inside this window, the amplitudes of the *Norm function* were averaged over frequency generating a characteristic single-peaked function of which the maximum was defined as uni-directional velocity *υ*_A0_. For each individual measurement of a cycle, the harmonic mean of the two bi-directional velocities was calculated to correct for inclination angles between the probe and bone surface [28]. Unstable measurements for which the absolute difference between two opposite velocities was larger than 50 m s^−1^ were eliminated [15]. The final velocity *υ*_A0_ of a tibia specimen was calculated by averaging the peak values of the velocity histograms obtained for each cycle.

### Reference measurements

#### Micro-computed tomography—39 μm

Micro-computed tomography (μCT) with 39 μm isotropic voxel size was used to measure cortical thickness (*Ct.Th*_*μCT*_) and *vBMD*. The proximal epiphyses were removed with a hand saw to fit the frozen shaft specimens into a custom-made thermo-isolated plastic tube. The tube containing the specimen was filled with dry ice and scanned with the μCT system (VivaCT 80, Scanco Medical, Brüttisellen, Switzerland). Before scanning, the bone’s longitudinal axis was aligned with the rotation axis of the sample holder. The diameter of the field of view was 50 mm, allowing the imaging of the entire shaft cross-section. Source voltage and current were set at 70 kV and 114 μA, respectively. Five hundred projections were acquired over a range of 360° using an integration time of 200 m s. A filtered back-projection reconstruction was used to obtain stacks of 1850 slices with an isotropic voxel size of 39 μm. The gray values of the images were transformed into mgHA^.^cm^−3^ based on a calibration procedure provided by the scanner vendor. The bone region insonified by AT was extracted from the μCT stack (approximately 30 mm, equivalent to 795 slices) and first binarized using Otsu’s method [31]. After this, the cortical bone compartment was automatically segmented applying the algorithm proposed by Burghardt et al. [32]. The radius of the structuring element for morphological closing of the mask was set to 0.03 mm. A manual correction was needed for one sample which had the highest *Ct.Po*_*μCT*_ (22%). *vBMD* was defined as the mean mineralization value for all voxels in the cortical compartment at the medial portion of the tibia and above the medullary canal. Ct.Th was calculated in that region as the minimum distance between endosteal and periosteal surfaces [33].

#### Scanning acoustic microscopy

Scanning acoustic microscopy (SAM) provided the acoustic impedance (*Z*_*SAM*_) of the cortical bone matrix. Cross sections of approximately 20 mm thickness were extracted from the diaphysis, site-matched with the region of the AT receiver array (Fig. 1b). Of each section, the proximal surface was polished using a planar grinder (Phoenix 4000, Buehler Ltd., Illinois) at constant speed (50 rpm) and decreasing grain size (ISO/FEPA grit: P80, P600, P1200, P2500, and P4000, Buehler Ltd., Illinois). Subsequently, the samples were washed and degassed for approximately 30 min. The custom-made microscope and scanning procedure have been described in detail elsewhere [34]. Briefly, a 100-MHz spherically focused transducer was used (KSI 100/60°, Krämer Scientic Instruments, Herborn, Germany) which had a − 6-dB bandwidth at the confocal pulse echo between 84.4 and 100.7 MHz. The − 6-dB depth of focus and lateral beam diameter in the focal plane were 139 and 19.8 μm, respectively [35]. The samples were immersed in a temperature-controlled tank with a 25 °C degassed 1% PBS solution. Images were acquired by moving the transducer along the x-y-plane with a scan increment of 12 μm. The scan time was up to 5 h. A defocus correction was applied before the images were converted into acoustic impedance maps (Fig. 3a) using calibration materials (PMMA and titanium). The cortical compartment was obtained by drawing the endosteal boundary manually (following the rules proposed by Malo et al. [10]) whereas the periosteal boundary was detected automatically by morphological region filling and tracing of the contour on the segmented image. Segmentation was performed using an adaptive threshold as described by Lakshmanan et al. [36]. The acoustic impedance (*Z*_*SAM*_) was calculated as the mean impedance value of all bone tissue pixels within the cortical compartment at the medial portion of the tibia and above the medullary canal. *Z*_*SAM*_ was converted into the stiffness coefficient *c*_*33*_ using a non-linear regression function [37].

#### Micro-computed tomography—7.4 μm

Rectangular parallelepiped samples of cortical bone were harvested from the cross sections, previously scanned with SAM (Fig. 3a), for the characterization of cortical porosity (*Ct.Po*_*μCT*_). The typical sample size was 2 × 3 × 4 mm^3^. Cutting was performed using a precision linear saw (Isomet 4000, Buehler GmbH, Düsseldorf, Germany). In the desktop μCT system (Skyscan 1172, Bruker MicroCT, Kontich, Belgium), the samples were positioned so that the anatomical vertical axis was aligned with the rotation axis of the sample holder. A source voltage of 80 kV, a current of 100 μA, and steps of 0.3° over 180° rotation were used. The exposure time for each frame was 320 ms. Twenty frames were averaged leading to a total scan time of 60 min per sample. A 0.5-mm-thick aluminum filter reduced beam hardening artifacts. Images were saved as 16-bit TIFF files and reconstructed using a filtered back-projection algorithm (NRecon, V1.6.10.4, Skyscan NV, Kontich, Belgium) with 20% ring artifact correction. For each parallelepiped sample, a stack of 650 sections was reconstructed with a 1968 × 1968 pixel field of view and 7.4 μm isotropic voxel size. Further post-processing was performed using the software CTan (V1.16.1.0, Skyscan NV, Kontich, Belgium). To separate the sample from the background, a semi-automatic procedure was performed, based on manual contouring on a selected number of slices, and followed by interpolation. A Gaussian 2D filter (R = 1) was applied to the images which were then segmented using the Otsu’s method [31]. Finally, in a 3D analysis, the tissue volume (TV), pore volume (PV), and cortical porosity (Ct.Po = PV / TV * 100%) were calculated.

### Statistical analysis

The normality of the distributions of the derived parameters was verified with Shapiro-Wilk tests. Linear regression analysis and Pearson’s correlation coefficients were used to quantify the degree of association between parameters obtained from AT, SAM, and μCT. Bland-Altman plots were used to reveal biases in the prediction of *Ct.Po*_*μCT*_ and *Ct.Th*_*μCT*_. Differences between the means were tested either with paired *t* tests or Wilcoxon signed-rank tests in case the data was not normally distributed. Correlations were considered statistically significant for *p* values lower than 0.05. Stepwise multiple regression analysis was applied to evaluate the optimal combination of parameters to predict *Ct.Po*_*μCT*_ and *Ct.Th*_*μCT*_. The single-cycle repeatability of the AT measurement parameters (*Ct.Th*_*AT*_, *Ct.Po*_*AT*_, *υ*_*FAS*_, *υ*_*A0*_) was estimated using the root-mean-square average of the standard deviation [38] obtained from at least two repeated cycles. Unless stated otherwise, all image processing and statistical analysis were performed using MATLAB (R2017a, The MathWorks Inc., Natick, MA, USA).

## Results

The results from 17 out of 19 specimens were used for statistical analysis. Two samples (Fig. 5a, b) were excluded due to large deviations of the ultrasonic measurements between the cycles. The distributions of the parameters were normal after logarithm transformation except for *vBMD* (*p* = 0.004). Between both groups, no statistically significant differences were found for all parameters. The single-cycle repeatability was 0.32 mm for *Ct.Th*_*AT*_, 2.9% for *Ct.Po*_*AT*_, 43.3 m^.^s^−1^ for *υ*_*FAS*_, and 47.8 m^.^s^−1^ for *υ*_*A0*_. Table 1 shows the results and correlations between the different measurement parameters.

**Table 1:** Descriptive statistics: mean, standard deviation (SD), and range of the measurement variables. R^2^ of the univariate linear regression between the variables. The outlier (Fig. 4 circle) has not been removed. The associations are positive unless otherwise indicated by a negative sign. *n.s.* not significant. *N* = 17

	Descriptive statistics		R^2^		
	Mean ± SD	Range	Ct.Po_μCT_	Ct.Th_μCT_	vBMD
Ct.Po_AT_ (%)	11.1 ± 7.7	2.0–25.0	0.83^***^	n.s.	(−) 0.80^***^
Ct.Th_AT_ (mm)	4.0 ± 0.6	2.9–5.2	n.s.	0.57^***^	n.s.
υ_FAS_ (m^.^s^−1^)	3806 ± 183	3429–4034	(−) 0.49^**^	n.s.	0.58^***^
υ_A0_ (m^.^s^−1^)	1701 ± 89	1583–1865	(−) 0.72^***^	0.28^*^	0.64^***^
Ct.Po_μCT_ (%)	11.5 ± 5.2	5.6–22.8	–	0.27^*^	0.77^***^
Ct.Th_μCT_ (mm)	3.6 ± 0.8	2.0–5.1	0.27^*^	–	n.s.
vBMD (g^.^cm^−3^)	923 ± 59	794–980	0.77^***^	n.s.	–
Z_SAM_ (MRayl)	6.7 ± 0.5	6.8–8.4	n.s.	n.s.	n.s.
c_33_ (GPa)	32.0 ± 3.5	24.3–36.7	n.s.	n.s.	n.s.

*p < 0.05; **p < 0.01; ***p < 0.001

### Prediction of cortical porosity

**Fig. 4:**
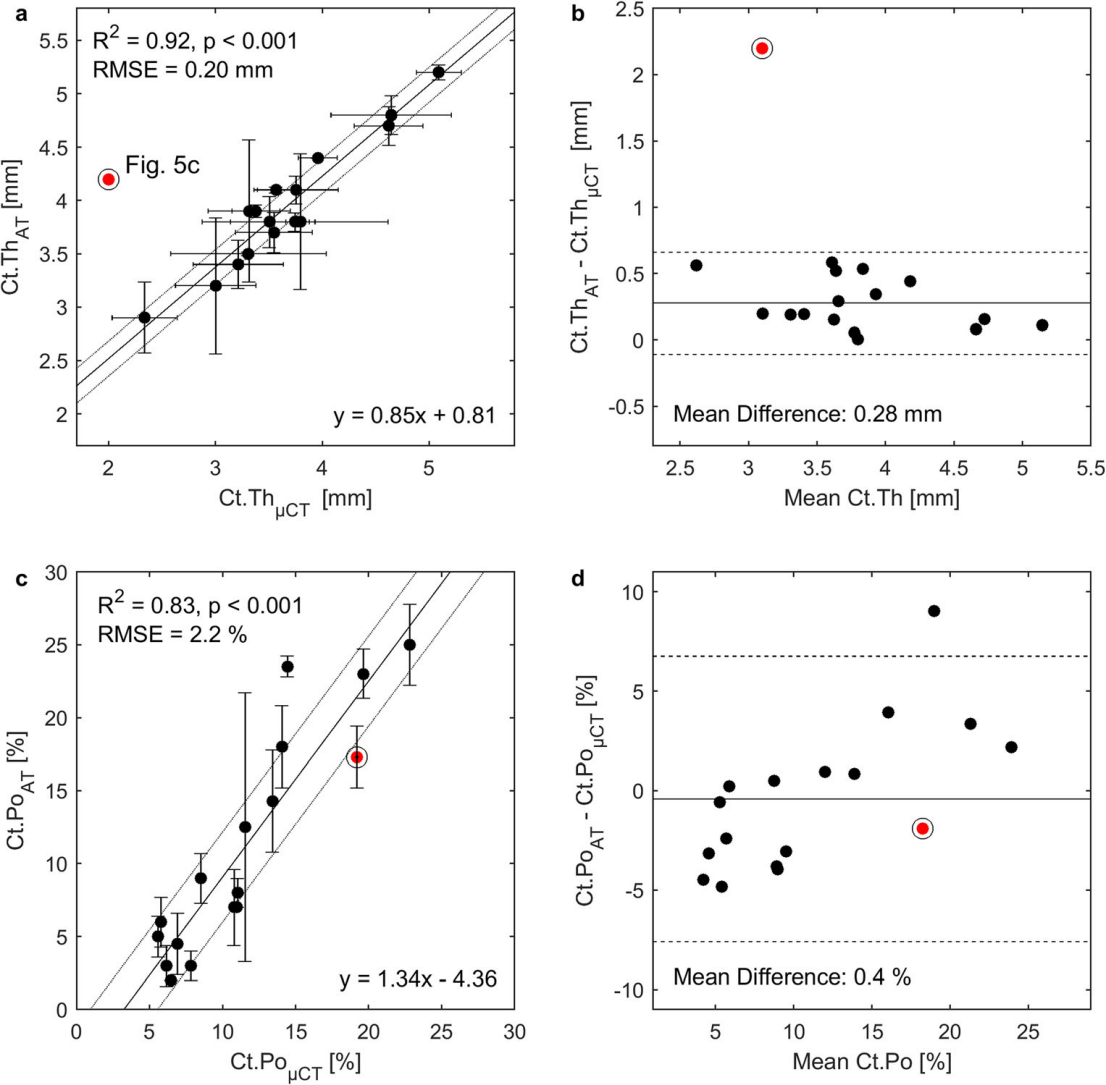
**a** Correlation between cortical thickness estimated from ultrasound axial transmission (*Ct.Th*_*AT*_) and micro-computed tomography (*Ct.Th*_μCT_) with 39 μm voxel size after exclusion of one outlier (red circled) (Fig. 5c). The correlation including the outlier was R^2^ = 0.57, *p* < 0.001, RMSE 0.37 mm. Horizontal error bars represent sample-specific Ct.Th variations in the region below the probe obtained from the full width of the distance histogram at 60% of its maximum. Vertical error bars represent standard deviations (within at least two cycles). **b** Mean difference and lines of according Bland-Altman plot were calculated without outlier. Mean difference including the outlier was 0.39 mm and 0.28 mm excluding the outlier. **c** Correlation between cortical porosity from AT (*Ct.Po*_*AT*_) and μCT with 7.4 μm voxel size (*Ct.Po*_*μCT*_). **d** According Bland-Altman plot. Solid lines represent fitted linear regression curves (**a**, **c**) and *mean* values (**b**, **d**). Dotted lines in (**a**, **c**) represent RMSE. Dashed lines and in (**b**, **d**) indicate 95% confidence intervals at ± 1.96 SD. *N* = 17

The best predictor for cortical porosity (*Ct.Po*_*μCT*_) was *Ct.Po*_*AT*_ (R^2^ = 0.83, *p* < 0.001, RMSE = 2.2%, Fig. 4c). The difference between the estimates of the two methods was not statistically significant. Figure 4d shows the according Bland-Altman plot which suggests a bias of Ct.Po that depends positively on the porosity level. This effect was also reflected in the slope of the linear regression (Fig. 4c) which, however, was not statistically different from 1 (confidence interval 1.00–1.68).

### Prediction of cortical thickness

**Fig. 5:**
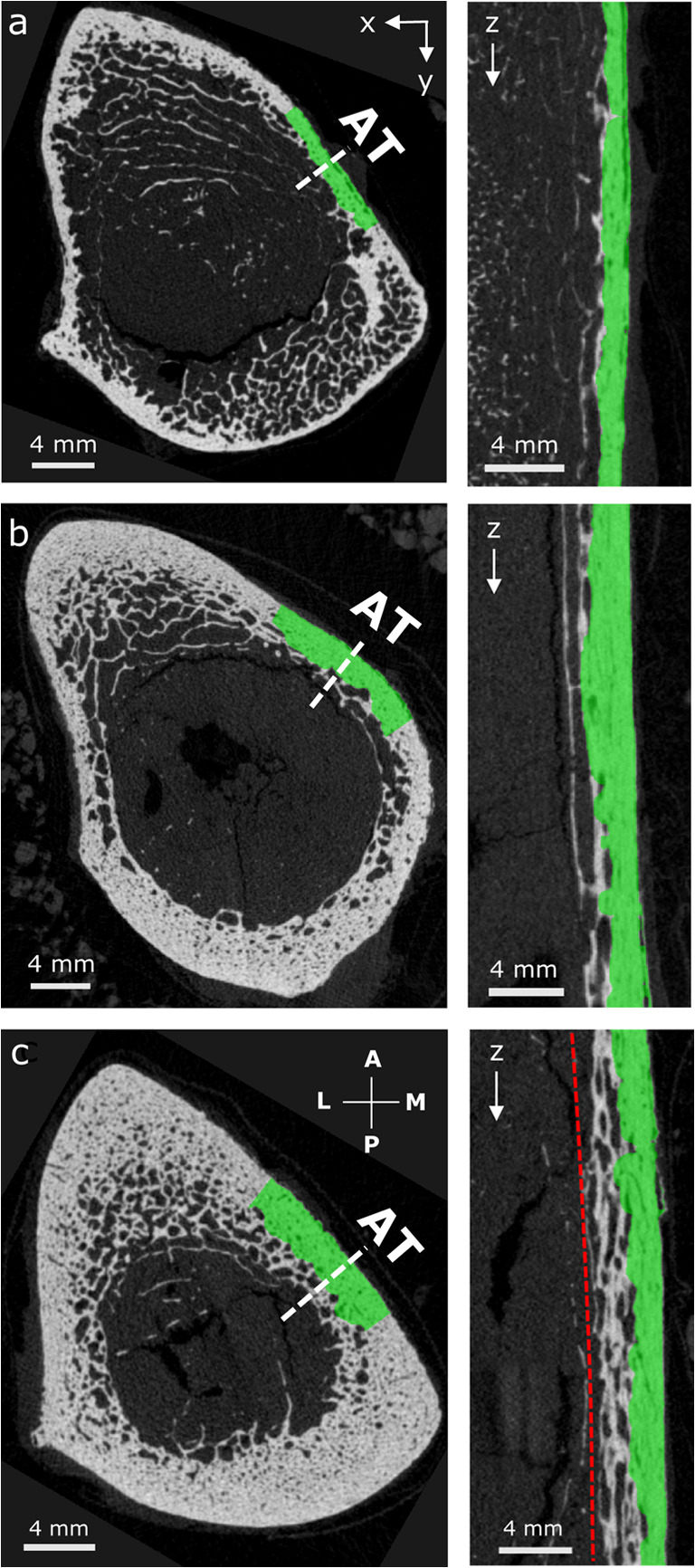
Images obtained from μCT stacks of 795 slices with 39 μm isotropic voxel size. Longitudinal sections (right) were taken at the dashed line in the cross sections (left) where axial transmission (AT) was performed. The segmented cortex mask, used to calculate site-matched *vBMD* and *CtTh*_*μCT*_, is shown in green. **a**, **b** AT failure cases. **c** Outlier sample with heavily trabecularized cortex (indicated by a circle in Fig. 4). Here, the measurement of a reference *CtTh*_*μCT*_ (green) does not agree with *CtTh*_*AT*_ (red line). The cross sections were rotated according to the anatomical alignment: A anterior, M medial, P posterior, and L lateral

*Ct.Th*_μCT_ was best predicted by *Ct.Th*_*AT*_ (R^2^ = 0.92, *p* < 0.001, RMSE = 0.20 mm) after removal of one sample with a heavily trabecularized cortex (Fig. 5c). For this sample, the difference between the two Ct.Th estimates was particularly large (2.2 mm); approximately five times larger than the 95% confidence interval at ± 1.96 SD (0.4 mm, Fig. 4b). Figure 5c suggests that ultrasonic guided waves may also propagate in the trabecularized bone region (red line) which in the μCT images was not considered to belong to the cortical compartment (green). Moreover, AT significantly overestimated Ct.Th with respect to μCT (*p* < 0.001; mean difference between both methods 0.28 mm). The second best predictor of *Ct.Th*_*μCT*_ was *υ*_*A0*_ (R^2^ = 0.29, *p* = 0.031, RMSE = 0.59 mm). Multiple regression analysis did not perform better than the abovementioned simple regression analysis.

## Discussion

In this ex vivo study, the estimation of cortical thickness (Ct.Th) and porosity (Ct.Po) at the human tibia using full spectrum guided-wave analysis was successfully validated against site-matched high-resolution micro-computed tomography (μCT). We utilized a novel 500-kHz axial transmission (AT) transducer which was designed to optimize the excitation of guided wave modes at the diaphysis of the tibia. Furthermore, we accounted for a possible inter-specimen variation of the cortical bone matrix elasticity by incorporating the acoustic impedance from site-matched scanning acoustic microscopy (SAM). The variability of the matrix elasticity did not improve our model-based predictions of Ct.Po and Ct.Th. This result supports the concept of variations in matrix stiffness which has a minor impact on the effective elasticity tensor compared to the effect of variations in porosity [26]. Note that our matrix stiffness measurements might have also been biased by experimental errors. For the first time, the A_0_ mode velocity (*υ*_*A0*_) was measured in human cortical bone using SVD-enhanced 2D Fourier transforms and compared to site-matched Ct.Th and Ct.Po at the same time.

The systematic overestimation of Ct.Th (0.28 mm) by AT has twofold implications. On the one hand, the *Ct.Th*_μCT_ reference measurement is affected by the natural variability of the bone morphology, as illustrated in Fig. 5, and by the horizontal error bars of Fig. 4a. On the other hand, the exact behavior of guided waves in samples with irregular and trabecularized boundaries (Fig. 5c) has not yet been investigated. Interpreting the results for these cases is particularly challenging, since the distinction of cortical bone from the trabecular compartment in the μCT images is itself a matter of arbitrary decision, as discussed in the next paragraph. Numerical simulations of ultrasound propagation using realistic (structurally heterogeneous) cortical bone models could help in clarifying to what extent trabecularized regions participate in the waveguide. Figure 4d suggests a bias of Ct.Po that depends positively on the porosity level. This bias might be partially caused by larger partial volume effects in the estimation of reference *Ct.Po*_μCT_ for samples with higher Ct.Po. The assumption of a waveguide model with invariant matrix stiffness might also contribute to the bias. To partially correct for this effect, we accounted for variations in the axial tissue stiffness (c_33_) by means of average acoustic impedance of mineralized tissue from SAM. Future ex vivo studies could incorporate the full transverse isotropic stiffness tensor of the waveguide, e.g., as experimentally obtained from resonant ultrasound spectroscopy [39].

The prediction of Ct.Th (R^2^ = 0.57) was weaker than for Ct.Po (R^2^ = 0.83). This was mainly caused by one sample (indicated with a circle in Fig. 4) which had a heavily trabecularized cortex as shown in Fig. 5c. When this sample was excluded, the correlation between *Ct.Th*_*AT*_ and *Ct.Th*_μCT_ improved significantly (R^2^ from 0. 57 to 0.94, RMSE from 0.37 to 0.16 mm). We believe that this is due to the definition used for the determination of *Ct.Th*_*μCT*_, which is especially uncertain within highly trabecularized cortical bone regions. Note that a consensus on how to segment the cortical bone compartment has not yet been reached. The longitudinal μCT section of Fig. 5c (right) obtained from the outlier sample explains the Ct.Th discrepancy between μCT (green) and AT (red line). The figure suggests that guided waves also propagated in the trabecularized bone region, but our applied cortical compartment segmentation algorithm [32] did not include this region.

We have used cortical bone samples from adults without report of metabolic bone diseases. For this reason, we cannot conclude on the general applicability of our method to subjects with considerably different matrix stiffness compared to normal adult bone (e.g., children, patients with osteogenesis imperfecta [40], or patients on long-term bisphosphonate treatment [41]). To overcome the assumption of invariant matrix stiffness, the elastic tensor could be derived from the plate model instead of porosity as it was previously suggested [7, 42]. However, this approach would increase the number of unknown model coefficients and require complete resolutions of the experimental dispersion curves. Our current guided wave transducer technology is limited, particularly in spatial resolution, and therefore cannot yet provide such reconstruction quality.

The major limitation of this study was the small sample size used for statistics (*N* = 17). Nevertheless, a broad range of *Ct.Th*_μCT_ (2.3–5.1 mm) and *Ct.Po*_*μCT*_ (5.6–22.8%) was covered, which represents what is usually found in other studies [26, 43]. Furthermore, the dependency of *υ*_*FAS*_ on *vBMD* is consistent with previous studies at the tibia using different frequencies (200 kHz [14], 250 kHz [44], 400 kHz [45], and 1.25 MHz [43]). However, we did not find a statistically significant correlation between *υ*_*FAS*_ and *Ct.Th*_*μCT*_, as it has been observed for the tibia using 200 kHz [14] and 400 kHz [45]. The dependency of *υ*_*A0*_ on Ct.Th and *vBMD* confirms the findings of an ex vivo study at the radius using 200-kHz AT [21]. We excluded two samples due to large deviations of the ultrasonic measurements between the cycles. The one failure case (Fig. 5a) had a very thin cortical bone layer (Ct.Th < 2.0 mm) in which ultrasonic guided waves cannot sufficiently be excited using the 500-kHz probe. Alternatively, we could have used the 1-MHz probe which was originally designed for measurements at the thinner radius. The second failure case exhibited a very inhomogeneous and trabecularized cortex (Fig. 5b) which might not have guided the ultrasonic waves appropriately.

Previous studies which measured *υ*_*A0*_ in cortical bone extracted the wave packages of the A_0_ mode in the time domain [5, 19]. In contrast, our method isolates the A_0_ dispersion curve in the frequency-phase velocity domain. We assume that this approach is more accurate since it ensures that no other signals interfere. Furthermore, we accounted for small inclination angles between the probe and bone surface using bi-directional measurements which will become more beneficial in vivo in the presence of soft tissue. However, the in vivo applicability of this novel *υ*_*A0*_ measurement technique remains to be demonstrated.

A former data acquisition protocol, used by our group at the radius, was based on three cycles of ten successive measurements [42]. For the current work, we used notably longer scan times (i.e., 400 successive measurements per cycle) and slowly tilted the probe. In the post-processing, a waveguide model was then fitted to the dispersion curves of each measurement, providing estimates of Ct.Th and Ct.Po. When the dispersion curves were too noisy or incomplete, the solution to the problem was no longer unique, as indicated by several local maxima in the objective function. Therefore, we used a criterion that allowed us to exclude such problematic measurements. In the future, this automatic criterion could be evaluated in real time to retain only measurements without model ambiguities.

In conclusion, the best predictions of cortical thickness (Ct.Th) and porosity (Ct.Po) were obtained from a plate model with invariant matrix stiffness, which was fitted to the measured guided wave dispersion curves. The second best predictors of Ct.Po and Ct.Th were *vBMD* and *υ*_*A0*_, respectively. No further enhancements were observed by accounting for variations in matrix stiffness. Clinical pilot studies are currently ongoing to confirm the possibility of a full-spectrum ultrasonic guided-wave analysis in vivo.

## Electronic supplementary material

Online Resource 1 Fully automatic uni-directional A_0_ mode phase velocity (*υ*_A0_) calculation procedure. **a** SVD-enhanced 2D Fourier transform (*Norm function*) of the multi-dimensional (5 × 24) radio-frequency signals corresponding to all possible emitter-receiver pairs in the frequency-wavenumber (*f*-*k*) domain. **b** *Norm function* converted into frequency-phase velocity (*f*-*cp*) domain. The A_0_ mode is extracted using fixed frequency (0.5 to 0.8 MHz) and *c*_*p*_ ranges (1400 to 1900 m^.^s^−1^). **c** In that range, the amplitudes of the *Norm function* are averaged over frequency, generating a characteristic single-peaked function (**d**) of which the maximum is obtained (*υ*_A0_). (PNG 12732 kb)

High resolution image (TIF 12732 kb)
