# Supplementary figures and images for: Ex vivo cortical porosity and thickness predictions at the tibia using full-spectrum ultrasonic guided-wave analysis
Source: Arch Osteoporos. 2019 Feb 20;14(1):21. doi: 10.1007/s11657-019-0578-1 (PMC6394459; doi:10.1007/s11657-019-0578-1)

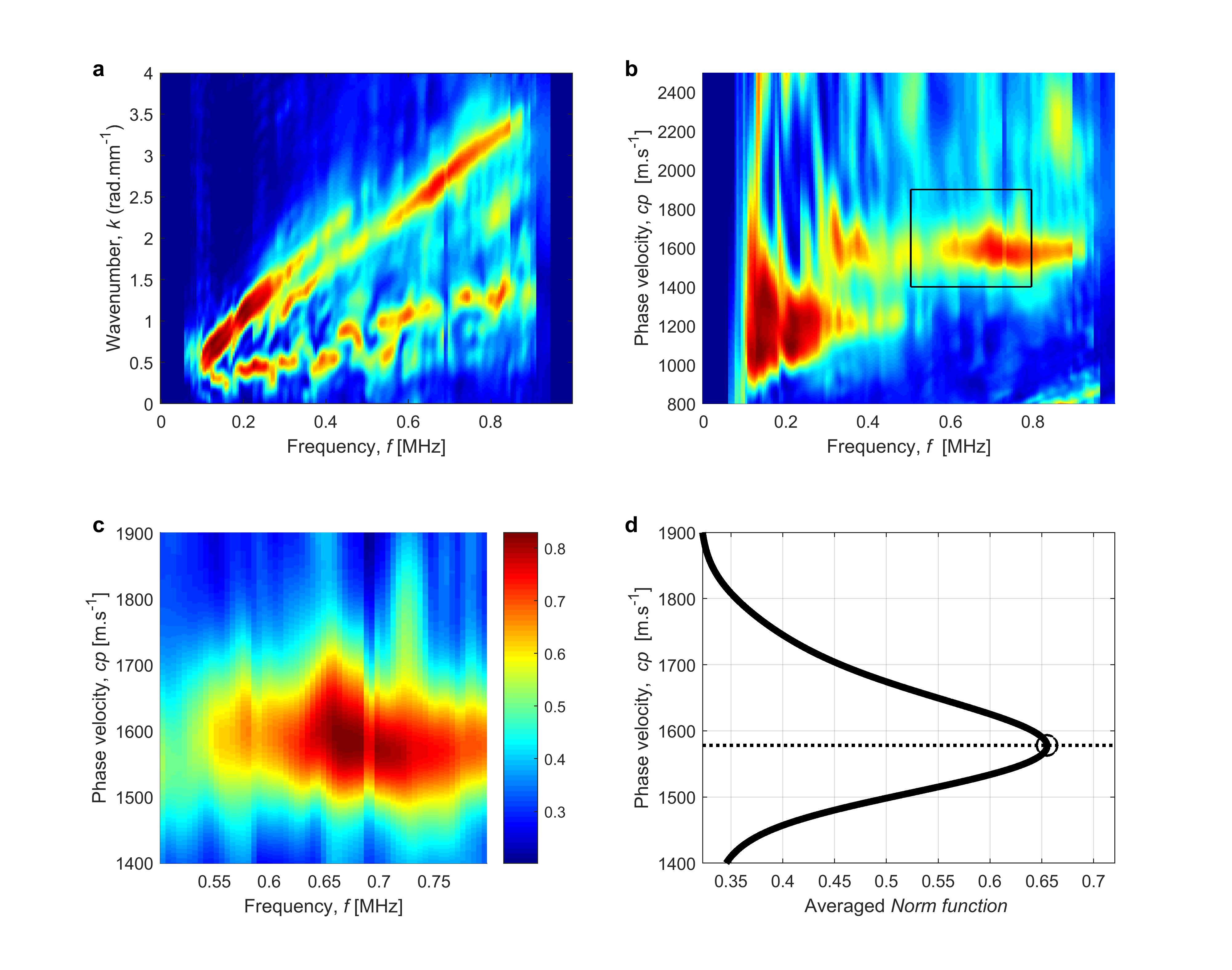

Supplement: Supplementary file 1 — Fully automatic uni-directional A0 mode phase velocity (υA0) calculation procedure. a SVD-enhanced 2D Fourier transform (Norm function) of the multi-dimensional (5 × 24) radio-frequency signals corresponding to all possible emitter-receiver pairs in the frequency-wavenumber (f-k) domain. b Norm function converted into frequency-phase velocity (f-cp) domain. The A0 mode is extracted using fixed frequency (0.5 to 0.8 MHz) and cp ranges (1400 to 1900 m.s−1). c In that range, the amplitudes of the Norm function are averaged over frequency, generating a characteristic single-peaked function (d) of which the maximum is obtained (υA0). (PNG 12732 kb) [file 11657_2019_578_Fig6_ESM.png]
